# Activation of cGAS-STING signaling in senescent cells promotes the aging process by remodeling the functions of the immune system

**DOI:** 10.1007/s10522-025-10353-5

**Published:** 2025-11-16

**Authors:** Antero Salminen, Kai Kaarniranta, Anu Kauppinen

**Affiliations:** 1 Department of Neurology, Institute of Clinical Medicine, University of Eastern Finland, P.O. Box 1627, FI-70211 Kuopio, Finland; 2 Department of Ophthalmology, Institute of Clinical Medicine, University of Eastern Finland, P.O. Box 1627, FI-70211 Kuopio, Finland; 3 Department of Ophthalmology, Kuopio University Hospital, P.O. Box 100, FI-70029 KYS Kuopio, Finland; 4 School of Pharmacy, Faculty of Health Sciences, University of Eastern Finland, P.O. Box 1627, FI-70211 Kuopio, Finland

**Keywords:** Ageing, Cellular senescence, cGAS-STING, Immunosuppression, Immunosenescence, Inflammaging

## Abstract

An accumulation of senescent cells within tissues is a hallmark of the aging process. Cellular senescence is associated with an increased level of cytosolic dsDNA which primarily originates from a leakage of mitochondrial DNA (mtDNA) and a loss of genomic DNA integrity. Cytosolic dsDNA is an important alarming factor for cytosolic dsDNA sensors which trigger the remodeling of the immune system through diverse signaling pathways. The cyclic GMP-AMP synthase (cGAS)-stimulator of interferon genes (STING) (cGAS-STING) signaling is a major defence mechanism induced by an accumulation of cytosolic dsDNA in senescent cells. The cGAS-STING pathway stimulates immune responses via the interferon regulatory factor 3 (IRF3) and nuclear factor-κB (NF-κB)-driven pathways. The activation of cGAS-STING signaling in senescent cells generates pleiotropic immune responses in a context-dependent manner. For instance, cGAS-STING signaling induces proinflammatory responses by enhancing the secretion of cytokines, chemokines, and colony-stimulating factors. The secretion of many chemokines and colony-stimulating factors can remodel hematopoiesis and enhance thymic involution with aging. Moreover, cGAS-STING signaling promotes proinflammatory responses by stimulating the NLRP3 inflammasomes. On the other hand, cGAS-STING signaling aids in the resolution of inflammation by recruiting immunosuppressive cells into tissues and suppressing the pathogenic activity of T helper 17 cells. In addition, an increased cGAS-STING signaling in senescent cells stimulates the expression of inhibitory immune checkpoint ligands, such as PD-L1, and thus prevents their elimination by immune cells. Recent studies have clearly revealed that cGAS-STING signaling not only induces cellular senescence but it can also promote the aging process.

## Introduction

There is convincing evidence that cellular senescence is not only associated with the aging process and age-related diseases but many acute and chronic insults can also trigger the senescence state in cells within tissues (He and Sharpless 2017; Yousefzadeh et al. 2020). An arrest of the cell cycle and the appearance of a proinflammatory state, generally called the senescence-associated secretory phenotype (SASP), are the two major hallmarks of cellular senescence (Hernandez-Segura et al. 2018). This kind of age-related cellular senescence seems to be a tissue specific process as clearly revealed during physiological and accelerated aging of mice (Yousefzadeh et al. 2020). It does seem that cellular senescence is a driver of the aging process rather than a consequence of age-related pathological alterations. Several investigations have also revealed that senescent cells are able to expand the senescence state into neighbouring cells within tissues (da Silva et al. 2019). This bystander senescence is induced by the secretion of many SASP factors from senescent cells, such as reactive oxygen species (ROS) and proinflammatory cytokines. Importantly, senescent cells can regulate many functions of the immune system via the secretion of diverse SASP factors, e.g., colony-stimulating factors (CSF), cytokines, and chemokines, which can also remodel hematopoiesis and enhance myelopoiesis with aging (Elias et al. 2017; Gnani et al. 2019; Ho et al. 2019). It seems that the SASP factors released from senescent cells have a major role in the generation of the inflammaging state in aged humans.

**Fig. 1:**
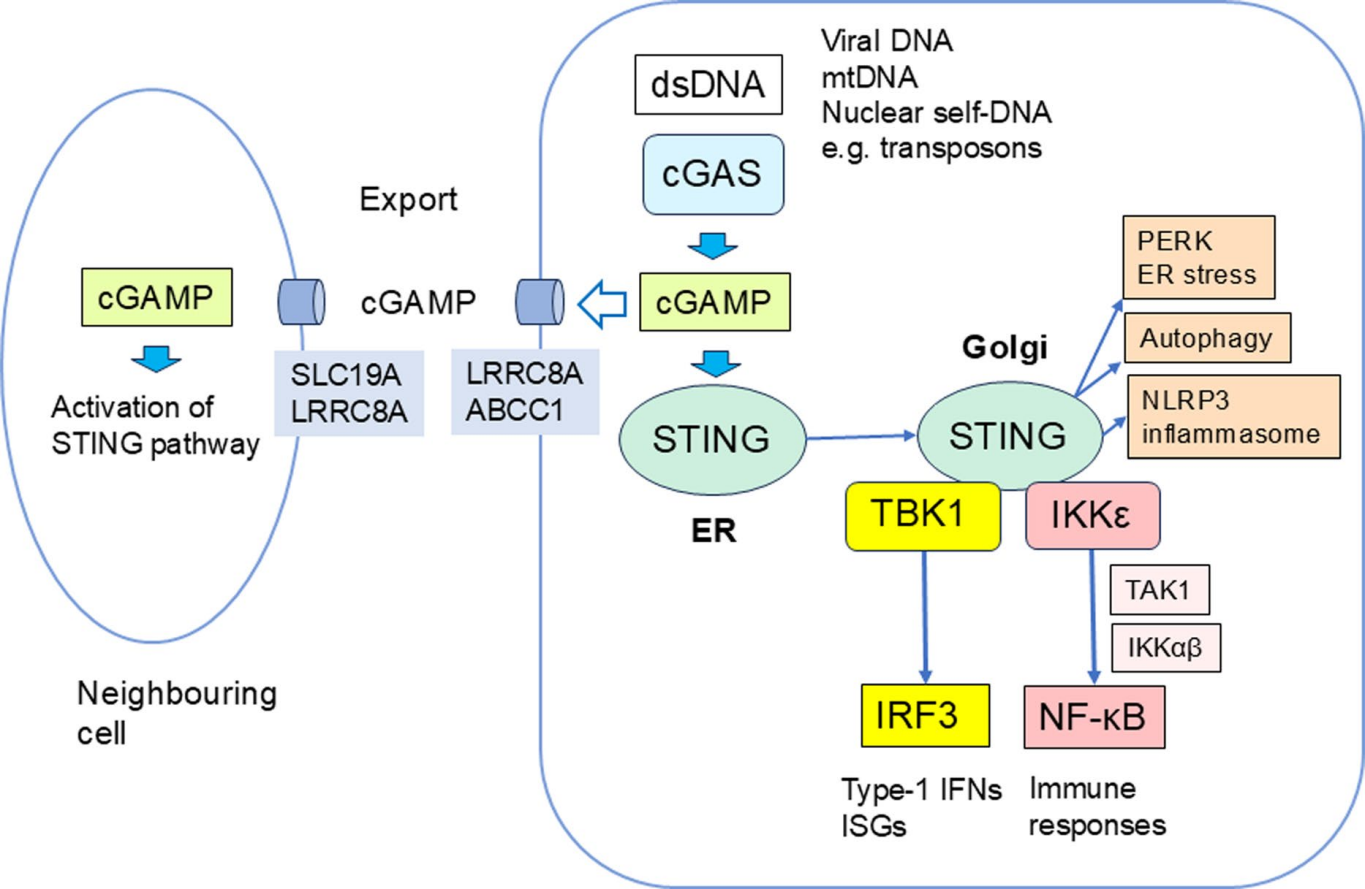
Schematic presentation of the cGAS-STING signaling pathway. Cytosolic dsDNA released from mitochondria, nuclei, or viruses stimulates the cGAS enzyme which generates cGAMP dinucleotides. Subsequently, these cGAMP dinucleotides stimulate the STING protein in the ER from which it will be transported into the Golgi complex where it forms the STING signaling platform. The cGAMP molecules can also be exported from the cell and from the extracellular space they can be imported into neighbouring cells where they activate STING signaling. The STING platform can interact with TBK1 and IKKε kinases which trigger downstream signaling pathways via IRF3 and NF-κB transcription factors. TBK1 activates the IRF3 factor which subsequently stimulates the expression of type-1 IFNs and many other ISGs. IKKε activates the TAK1 kinase which subsequently activates the IKKα and IKKβ kinases. The IKKα/β enzymes stimulate NF-κB signaling which is a major regulator of many immune responses. It is known that the STING platform can also stimulate non-canonical pathways, i.e., immune responses via NLRP3 inflammasomes, autophagy and ER stress by PERK signaling. Abbreviations: ABCC1, ATP binding cassette subfamily C member 1; cGAMP, cyclic guanosine monophosphate–adenosine monophosphate dinucleotide; cGAS, cyclic GMP-AMP synthase; dsDNA, double-stranded DNA; ER, endoplasmic reticulum; IFN, interferon; IKK, IκB kinase; IRF3, interferon regulatory factor 3; LRRC8A, leucine rich repeat containing 8 VRAC subunit A; mtDNA, mitochondrial DNA; NF-κB, nuclear factor-κB; NLRP3; NLR family pyrin domain containing 3; PERK, protein kinase R-like ER kinase; SLC19A, solute carrier family 19 member A; STING, stimulator of interferon genes; TAK1, transforming growth factor-β-activated kinase 1

Currently, it is known that the accumulation of cytosolic double-stranded DNA (dsDNA) has a crucial role in the generation of the senescence state in diverse cell types in many experimental conditions as well as in aged tissues (Glück et al. 2017; Lan et al. 2019; Takahashi et al. 2018). There are different intracellular sources from which dsDNA can be released into the cytoplasm. For instance, mitochondrial dysfunction can trigger the release of mitochondrial DNA (mtDNA) into the cytoplasm in both immune and non-immune cells (Zhong et al. 2022; Newman and Shadel 2023; Jimenez-Loygorri et al. 2024). In addition, many stresses, such as the aging process and many diseases, disturb the integrity of nuclear DNA inducing the release of fragmented dsDNA as well as retrotransposons into the cytosol (Dou et al. 2017; Lan et al. 2019; Mathavarajah and Dellaire 2024). The role of cytosolic dsDNA as an important alarming factor is emphasized by many observations indicating that both immune and non-immune cells possess (i) dsDNA sensors which can recognize cytosolic dsDNA (Hopfner and Hornung 2020) and (ii) enzymes which degrade cytosolic dsDNA and thus attenuate immune responses (Takahashi et al. 2018). The cyclic GMP-AMP synthase (cGAS)-stimulator of interferon genes (STING) (cGAS-STING) system is a major canonical defence mechanism against cytosolic dsDNA (Hopfner and Hornung 2020; Zhang et al. 2025) (Fig. 1). Interestingly, the activation of the cGAS-STING pathway not only induces protective changes in senescent cells but it also evokes the SASP state which stimulates inflammatory and immunosuppressive responses thus remodeling the properties of the immune system during the aging process. Next, we will briefly introduce the pathways driven by cGAS-STING signaling in senescent cells and then examine in detail how the chronic activation of the cGAS-STING defence system in senescent cells can remodel immune responses and thus induce immunosenescence and promote the aging process.

## cGAS-STING signaling pathways

Cytosolic dsDNA, derived either from genotoxic and mitochondrial stresses or bacterial and viral infections, is a major threat to the maintenance of cellular homeostasis. The recognition of cytoplasmic dsDNA via the cGAS-STING mechanism has a bacterial origin which evolved during the metazoan phase of evolution (Patel et al. 2023). Interestingly, the components of the cGAS-STING pathway are highly conserved in vertebrates, i.e., (i) the cGAS sensor of dsDNA in the cytoplasm, (ii) the cyclic GMP-AMP (cGAMP) dinucleotide second messengers, (iii) the STING adaptor platform, and (iv) many STING interacting signaling molecules (Hopfner and Hornung 2020; Zhang et al. 2025) (Fig. 1). Currently it is known that cGAS-STING signaling has a crucial role in the pathogenesis of several diseases and many drug discovery projects are attempting to target the various components of the cGAS-STING pathway (Decout et al. 2021; Yu and Liu 2021). In 2024, Dr. Zhijian Chen was awarded the Lasker Prize for his discovery of the cGAS enzyme and its role in triggering innate immunity.

## Cytosolic dsDNA activates cGAS signaling and cGAMP production

The cGAS enzyme is located predominantly in the nucleus where it is inhibited by interaction with nucleosomal histones, thus preventing its autoreactivity (Michalski et al. 2020). Given that the human cGAS-nucleosome core particle complex is bound to the acidic patch of the histone H2A-H2B dimer and nucleosomal DNA (Kujirai et al. 2020), it has been speculated that the ubiquitination of H2A and H2B could release the cGAS enzyme in association with DNA damage and its repair process (de Oliveira Mann and Hopfner 2021). For example, Cho et al. (2024) demonstrated that binding of the double-strand break sensor protein MRE11 complex to the human nucleosomes displaced the cGAS protein from the nucleosomal acidic patch. Currently, the mechanisms releasing cGAS from chromatin still need to be clarified. The cGAS protein contains the nuclear export signal (NES) and it is translocated from nuclei to cytoplasm by the CRM1 exporter protein (Sun et al. 2021). In cytoplasm, the binding of dsDNA to the cGAS enzyme opens the docking sites for ATP and GTP in the enzyme which subsequently catalyzes the formation of cyclic guanosine monophosphate (GMP)-adenosine monophosphate (AMP) dinucleotide (cGAMP) (Hall et al. 2017). The 2’,3’-cGAMP dinucleotide is a potent second messenger which activates the STING signaling pathway and subsequently stimulates many immune responses (Fig. 1). The cGAS enzyme is also able to control a myriad of cellular functions, e.g., it regulates senescence, autophagy, apoptosis, proliferation, metabolism, DNA replication and repair, as well as innate and adaptive immunity (Liu et al. 2022).

The activity of the cGAS enzyme is stringently regulated not only transcriptionally but especially via post-translational modifications and protein–protein interactions (Yu and Liu 2021; Zhang et al. 2025). The post-translational modifications include acetylation, phosphorylation, ubiquitination, and sumoylation, which can regulate the activity and function of the cGAS protein (Yu and Liu 2021). There are also several co-sensors which enhance the binding of the cGAS protein to dsDNA. For example, the Zinc finger CCHC-type containing 3 (ZCCHC3), Z-DNA binding protein 1 (ZBP1), and GTPase-activating protein SH3 domain-binding protein 1 (G3BP1) are major co-sensors and enhancers of dsDNA binding of the cGAS protein (Lian et al. 2018; Liu et al. 2019; Lei et al. 2023). It is known that the ZCCHC3 protein directly binds to dsDNA and enhances the recruitment of the cGAS protein to dsDNA (Lian et al. 2018). Furthermore, the ZBP1 protein is a sensor for Z-DNA released from mitochondria and it recruits the cGAS protein to this complex, which can subsequently activate STING signaling (Lei et al. 2023). ZBP1 has an important role in mitochondrial stresses inducing the release of mtDNA, e.g., in cardiotoxicity (Lei et al. 2023). The G3BP1 protein is a multipotent protein which can not only enhance the binding of dsDNA to the cGAS protein (Liu et al. 2019) but also assemble stress granules in cytoplasm (Yang et al. 2020). Moreover, the activity of cGAS-STING signaling can be suppressed by certain enzymes, e.g., (i) the three prime repair exonuclease 1 (TREX1) degrades cytosolic dsDNA and thus it impedes the activation of the cGAS enzyme (Takahashi et al. 2018; Lim et al. 2024), (ii) several members of the caspase family can directly cleave the cGAS enzyme (Xiong et al. 2021), and (iii) the transmembrane ectonucleotide pyrophosphatase/phosphodiesterase 1 (ENPP1) which cleaves extracellular cGAMP thus inhibiting many functions of this intercellular immunotransmitter (Wang et al. 2023). In fact, TREX1 and ENPP1 are considered as innate immune checkpoints.

The cGAMP dinucleotide targets the STING adaptor protein in the endoplasmic reticulum (ER) (Hopfner and Hornung 2020; Zhang et al. 2025) (Fig. 1). The STING proteins are stored in the ER as inactive dimers, but the binding of cGAMP induces a conformational change in the STING dimers leading to the formation of tetramers and higher-order oligomers (Shang et al. 2019). Subsequently, these oligomeric STING complexes are transported in the coat protein complex-II (COP-II) vesicles from the ER into the Golgi complex where STING proteins act as a platform structure interacting with different signaling molecules to stimulate the IRF3 and NF-κB pathways (Taguchi et al. 2021; Zhang et al. 2025). Interestingly, the cGAMP dinucleotides are not only intracellular second messengers but they can be exported out from affected cells and subsequently taken up by neighbouring cells where they can stimulate STING-driven signaling (Xie and Patel 2023) (Fig. 1). It is known that in mouse and human cells, the volume-regulated anion channels (VRAC/LRRC8) and the multi-drug ABCC1 transporters export the cGAMP dinucleotides across the plasma membrane (Zhou et al. 2020; Maltbaek et al. 2022). Accordingly, the solute carrier SLC19A1 and the anion channel LRRC8 can import the cGAMP dinucleotides into neighbouring cells and stimulate the STING/IRF3-mediated immune responses (Zhou et al. 2020; Luteijn et al. 2019) (Fig. 1). It seems that cGAMP can drive a paracrine regulation via STING signaling within tissues.

## The STING platform stimulates IRF3 and NF-κB signaling

The oligomeric STING platform represents a signaling hub in the Golgi complex linking different upstream and downstream signaling pathways (Coderch et al. 2023; Zhang et al. 2025). Binding of cGAMP to the STING dimer triggers its translocation to the Golgi and subsequent interaction with the TANK-binding kinase 1 (TBK1) (Balka et al. 2020; Zhang et al. 2025) (Fig. 1). Subsequently, TBK1 phosphorylates the STING protein which induces the recruitment and phosporylation of the IRF3 transcription factor. Next, the IRF3 factor is transported from the Golgi complex to the nuclei where it induces the transcription of the type-1 *IFN* genes. Finally, the IFNα/β cytokines promote the expression of a number of interferon-stimulated genes (ISG) via an activation of the IFN receptor 1 and 2 (IFNAR1/2)-mediated JAK/STAT signaling (Platanias 2005). In addition, the inhibitor of nuclear factor-κB kinase subunit epsilon (IKKε) can also bind to the STING platform. Balka et al. (2020) demonstrated that TBK1 and IKKε are redundant regulators which stimulate the STING-induced NF-κB responses in myeloid cells. In downstream, the TBK1/IKKε factors activate the TGF-β-activated kinase 1 (TAK1) which subsequently can trigger IKKα/β kinases and stimulate the canonical NF-κB signaling pathway in many cell types (Abe and Barber 2014; Balka et al. 2020; Balka and De Nardo 2021) (Fig. 1). It is known that the responses of the STING platform are closely connected to the energy metabolic state and the level of ER stress within the cell (Zhao et al. 2018; Luo et al. 2024). As described below, the STING-induced IRF3 and NF-κB signaling pathways have a major role in the regulation of both pro-inflammatory and immunosuppressive responses.

## The STING platform is associated with many non-canonical functions

Interestingly, the cGAS receptor is not the single sensor of DNA integrity which targets the STING platform (Unterholzner 2013; Dunphy et al. 2018; Coderch et al. 2023). For instance, the IFN-γ-inducible protein IFI16, also known as the IFN-inducible myeloid differentiation transcriptional activator, is a dsDNA-binding protein which activates STING-mediated immune responses in a cGAS-independent manner (Dunphy et al. 2018; Choubey 2022). Dunphy et al. (2018) demonstrated that IFI16 and the ataxia-telangiectasia mutated (ATM) protein, a serine/threonine kinase which is activated by DNA breaks, stimulated the NF-κB pathway via the non-canonical STING signaling. In addition, there are two other sensors for cytosolic DNA, i.e., the DEAD box helicase DDX41 and the Z-DNA binding protein 1 (ZBP1), both of which target the STING platform (DeFilippis et al. 2010; Singh et al. 2022). For instance, in human skin fibroblasts an infection by human cytomegalovirus (CMV) stimulated the expression of the ZBP1 protein which induced the expression of IFN-β via STING/IRF3 signaling (DeFilippis et al. 2010). There are also two sensors for the cytoplasmic dsDNA which do not signal via the STING platform to induce immune responses, i.e., the absent in melanoma 2 (AIM2) and the toll-like receptor 9 (TLR9) proteins (Jin et al. 2012; Chan et al. 2015). It is not surprising that there are several DNA sensors because an appearance of cytosolic dsDNA represents a danger molecule of disturbances in cellular homeostasis. Many receptors probably have redundant functions, they act in different cell types, or they differ in their ligand specificity (Unterholzner 2013).

Given that the STING protein resides in the ER, there are many investigations which have revealed that the cooperation between STING protein and the protein kinase R-like endoplasmic reticulum kinase (PERK) represents an important non-canonical pathway in cGAS-STING signaling (Zhang et al. 2022; Wan et al. 2024) (Fig. 1). Zhang et al. (2022) demonstrated that after the binding of cGAMP to the STING protein, it interacted and directly activated the ER-located PERK in human HEK293 cells. Subsequently, PERK phosphorylated the eukaryotic initiation factor 2α (eIF2α) which arrested the global cap-dependent translation and it promoted the translation of certain specific survival proteins. The properties of the STING-PERK-eIF2α pathway seem to be independent of any activation of STING-IRF3 signaling. Instead, STING-PERK signaling played a pivotal role in the generation of damage-induced cellular senescence and bleomycin-induced fibrosis in mouse lungs (Zhang et al. 2022). It seems that the major function of the STING-activated PERK-eIF2α signaling is not involved in immune responses but it triggers cellular senescence and fibrosis as well as stimulates many survival responses (Kalinin et al. 2023).

There is a close interaction between STING signaling and autophagy via the non-canonical regulation (Fig. 1). For example, the activation of STING signaling by cGAMP stimulates directly autophagy in a manner independent of TBK1/NF-κB and IRF3 signaling (Gui et al. 2019). The STING platform in the Golgi complex can induce the lipidation of the microtubule-associated proteins 1A/1B light chain 3B (LC3B) which is known to be a crucial step in the formation of autophagosomes (Gui et al. 2019). Gui et al. (2019) demonstrated that cGAS-STING signaling stimulated autophagy in human cells via a lipidation mechanism which is dependent on WIPI2 and ATG5 but independent on the canonical ULK1/VPS34/Beclin pathway. They also reported that an activation of a non-canonical autophagy pathway was able to cleanse both cytosolic dsDNA and the STING platforms. Interestingly, Konno et al. (2013) revealed that cGAMP induced ULK1-mediated phosphorylation of the STING protein which subsequently suppressed the function of IRF3 but not that of NF-κB signaling. These results indicate that autophagy prevents the persistent activation of STING-driven innate immunity.

There is clear evidence that the cGAS-STING pathway can activate the function of the NLR family pyrin domain containing 3 (NLRP3) inflammasomes and stimulate the secretion of pro-inflammatory IL-1β and IL-18 cytokines (Fig. 1). It is known that NF-κB signaling enhances the priming of NLRP3 inflammasomes by stimulating the expression of the NLRP3 protein and the proforms of IL-1β and IL-18 cytokines (Bauernfeind et al. 2009). Moreover, the activation of STING signaling can induce the binding of the IRF3 protein to the promoter region of the *NLRP3* gene and subsequently increase the expression of the NLRP3 protein (Xiao et al. 2023). In addition, Wang et al. (2020) reported that STING proteins were able to recruit NLRP3 receptors to the ER and thus activated inflammasomes by deubiquitinating the NLRP3 proteins. All these observations indicate that cGAS-STING signaling can stimulate the activation of NLRP3 inflammasomes via different mechanisms and trigger inflammatory responses in a context-dependent manner.

## Termination of cGAS-STING signaling

The termination of cGAS-STING signaling is a crucial mechanism to regulate the function of the cGAS-STING pathway. Currently, it is known that there are two mechanisms to terminate cGAS-STING signaling, i.e., the STING protein can be transported from the Golgi complex (i) back to the ER via retrograde membrane trafficking pathway or (ii) to the endolysosomal compartment for degradation (Mukai et al. 2021; Taguchi et al. 2021; Balka et al. 2023). In the retrograde trafficking, the coat protein complex 1 (COP-1) involving the STING, Surf4, and α-COP proteins relocates the STING protein to the ER (Mukai et al. 2021). It is known that mutations in the *COPA* gene cause the COPA syndrome which is a rare interferonopathy causing autoimmune disorders. The presence of cGAMP, a STING ligand, prevents the formation of the STING/Surf4/α-COP complex and thus lengthens STING signaling (Mukai et al. 2021). An alternative pathway to terminate STING signaling is to transport the STING protein for degradation in the endosomal-lysosomal compartment. Recent studies have revealed that the endosomal sorting complexes required for transport (ESCRT9) proteins encapsulate the ubiquitinated STING protein into the Lamp1-positive vesicles which transport the STING protein into autophagic degradation (Balka et al. 2023; Kuchitsu et al. 2023). Moreover, the cGAS protein can be ubiquitinated and targeted to the p62 protein-mediated autophagic degradation, thus inhibiting the activity of cGAS-STING signaling (Chen et al. 2016). The above mentioned studies also revealed that the inhibition of the autophagic degradation of either the cGAS or the STING proteins promotes immune responses.

## Activation of cGAS-STING signaling is associated with cellular senescence

**Fig. 2:**
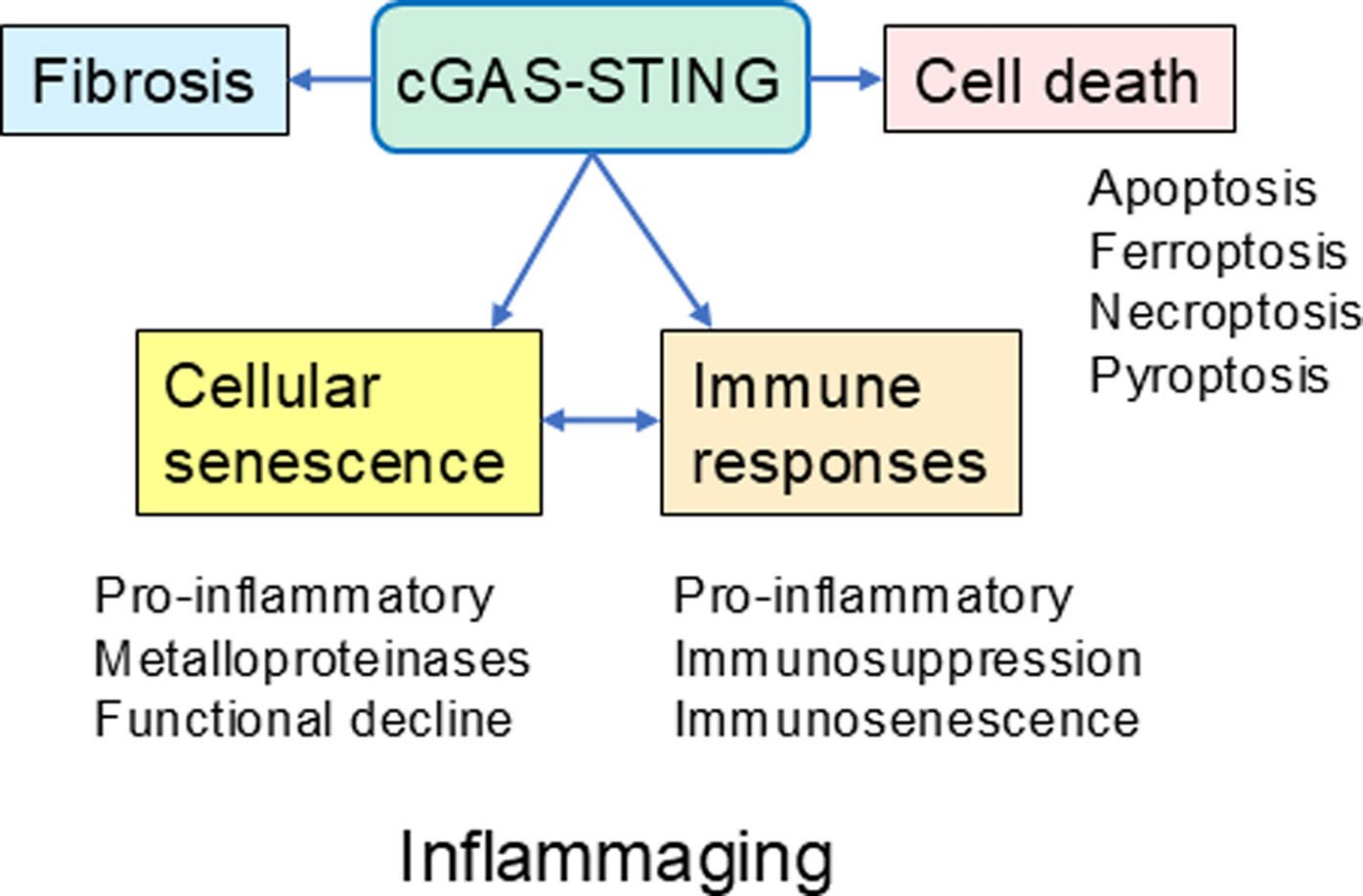
cGAS-STING signaling promotes general hallmarks of the aging process

There is robust evidence that cellular senescence is associated with an increased amount of cytosolic dsDNA in both cultured cells and in aged tissues (Glück et al. 2017; Takahashi et al. 2018; De Cecco et al. 2019; Lan et al. 2019). Given that cellular senescence increases with aging within tissues, this is consistent with many observations that there exist disturbances in mitochondrial homeostasis as well as a loss of genomic DNA integrity and a cytoplasmic accumulation of transposons which all increase the level of cytosolic dsDNA and promote cellular senescence (Niedernhofer et al. 2018; De Cecco et al. 2019) (Fig. 2). The role of cytosolic dsDNA as a cause of cellular senescence has been underscored by many investigators, e.g., an impaired activity of cytoplasmic DNAases, such as TREX1 and Dnase 2, significantly increased the amount of cytosolic dsDNA and induced a cellular senescence state with many characteristics of SASP (Takahashi et al. 2018; Du et al. 2023; Techer et al. 2024). Moreover, it is known that a deficiency in mitophagy, a hallmark of the aging process, increased the cytosolic levels of mtDNA and subsequently stimulated cGAS-STING signaling and cellular senescence in different cell types (Zhong et al. 2022; Jimenez-Loygorri et al. 2024). Lan et al. (2019) demonstrated that replicative senescence induced an accumulation of cytosolic dsDNA within human fibroblasts. They also reported that cytosolic dsDNA stimulated the cGAS-STING-dependent cellular senescence and induced many proinflammatory properties. Moreover, the level of extranuclear DNA was clearly increased in skin fibroblasts isolated from Hutchinson-Gilford and Ataxia telangiectasia (AT) patients, well-known progeroid syndromes. Progeroid fibroblasts displayed several markers of cellular senescence, such as an increased secretion of IFN-α, TNF-α, and IL-6 cytokines. The fibroblasts from AT patients revealed a robust elevation in the levels of STING, TBK1, and IRF3 proteins, an indication of the activation of cGAS-STING signaling. In conclusion, there is convincing evidence that the accumulation of dsDNA into the cytoplasm stimulates cGAS-STING signaling which has a crucial role in cellular senescence.

Both the IRF3 and NF-κB signaling pathways are able to stimulate cellular senescence and subsequently induce diverse immune responses via the secretion of many SASP factors (Salminen et al. 2012; Wang et al. 2024) (Fig. 1). For example, the activation of the IRF3 transcription factor stimulates both the expression and secretion of the IFN-α and IFN-β cytokines. Several studies have indicated that the IRF3 factor is a potent inducer of cellular senescence, either by a direct transactivation of the *p53* and *RB* genes (Moiseeva et al. 2006; Wu et al. 2024) or via signaling through the IFNAR pathway induced by IFN-α and IFN-β (Frisch and MacFawn 2020; Wang et al. 2024). The p53 and RB proteins arrest cell growth and silence certain genes, whereas IFN-α and INF-β are important SASP components and thus they can induce many immune responses. The IFN-α/β cytokines activate IFNAR/JAK1 signaling and subsequently they induce the expression of the IFN-stimulated genes (ISG) via stimulation of the STAT1/2 pathways (Schneider et al. 2014; Ji et al. 2023; Wang et al. 2024). For instance, the type-1 IFNs have an important role in the generation of cell-cycle arrested senescent cells by immunoediting stem cells during the developmental process (Yu et al. 2015) or by inhibiting the growth of tumor cells (Brenner et al. 2020). Currently, the role of type-1 IFNs in cellular senescence needs to be clarified.

The NF-κB signaling system is not only a major inducer of many SASP properties within senescent cells but it is also a master regulator of diverse responses in the immune system (Li and Verma 2002; Rovillain et al. 2011; Salminen et al. 2012; Capece et al. 2022). cGAS-STING signaling is one of many mechanisms which are able to stimulate NF-κB signaling although with aging, it has an important role in the dsDNA-induced remodeling of the immune network. Interestingly, it is known that the activity of NF-κB signaling is significantly upregulated with aging in many tissues (Helenius et al. 1996, 2001), i.e., simultaneously with an increased accumulation of both dsDNA and senescent cells into aged tissues. The activation of NF-κB signaling is a crucial transcriptional regulator of the expression of many proinflammatory cytokines, chemokines, and colony-stimulated factors (CSF) secreted by senescent cells although NF-κB signaling also promotes the expression of many anti-apoptotic factors as well as cell-cycle and metabolic regulators (Salminen et al. 2008; Freund et al. 2010; Capece et al. 2022). In addition, the NF-κB pathway is an important regulator of many processes of adaptive immunity, immunosenescence, and the aging process (Salminen et al. 2008; Bektas et al. 2017; Capece et al. 2022). There are several investigations indicating that the activation of STING signaling by DNA damage promotes both senescence and age-related degeneration via the NF-κB pathway (Guo et al. 2021; Zhang et al. 2021). For instance, Tilstra et al. (2012) demonstrated that the inhibition of NF-κB signaling delayed cellular senescence induced by DNA damage in the mouse XFE progeroid syndrome. Accordingly, Zhang et al. (2021) reported that an inhibition of NF-κB signaling reduced the level of markers of cellular senescence in many tissues of the Ercc1 and Zmpste24 progeroid mice which are recognized models of an accelerated aging process induced by DNA damage. These two reports also indicated that inhibition of NF-κB signaling significantly reduced the severity of age-related degeneration within the tissues of progeroid mice. Moreover, it is known that chronic activation of STING signaling robustly stimulated NF-κB-dependent secretion of the CXCL2 chemokines which subsequently promoted the recruitment of immunosuppressive myeloid-derived suppressor cells (MDSC) into mouse tumors (Nambiar et al. 2023). It is known that immunosuppressive cells can induce age-related senescence of diverse immune cells both in the aging process and age-related diseases (Salminen 2021). Interestingly, recent studies have revealed that cGAS-STING signaling has an important role in the generation of immunosenescence in diverse immune cells (Schmitz et al. 2023). It seems that the activation of cGAS-STING signaling not only induces cellular senescence in non-immune cells but it also triggers immunosenescence (Fig. 2).

## cGAS-STING signaling remodels the immune network

The major role of the cGAS-STING signaling pathway is to alert the immune system to combat pathogen infections as well as diverse disturbances in the maintenance of tissue homeostasis induced by a loss of DNA integrity. The activation of the cGAS-STING pathway remodels the immune network through its multifaceted effects on innate and adaptive immunity (Fig. 2). The cGAS-STING signaling pathway not only induces proinflammatory responses but it can also affect the maturation of myeloid and lymphoid cells in the bone marrow and thymus (Liao et al. 2020; Martinez and Hogquist 2023). cGAS-STING signaling can also evoke many anti-inflammatory and immunosuppressive properties, e.g., in the resolution phase of inflammation as well as striving to protect against the detrimental effects of chronic inflammation (Veldhuis et al. 2003; Damasceno et al. 2022; Chen et al. 2024). Moreover, cGAS-STING signaling has a crucial role in the course of chronic inflammatory diseases, e.g., in atherosclerosis (Sakai et al. 2023) and rheumatoid arthritis (Zhu and Zhou 2024). Several review articles have described the important role played by cGAS-STING signaling in the age-related chronic low-grade proinflammatory state (Paul et al. 2021; Yu et al. 2022; Gulen et al. 2023).

## cGAS-STING signaling promotes proinflammatory responses

The activation of cGAS-STING signaling in senescent cells produces a major part of the proinflammatory SASP factors via NF-κB signaling (Guo et al. 2021; Herbstein et al. 2024). For instance, the activation of the NF-κB pathway not only stimulates the expression of a number of cytokines, chemokines and CSFs but also adhesion molecules, cell cycle regulators and anti-apoptotic factors (Chien et al. 2011; Taniguchi and Karin 2018; Capece et al. 2022). It should be recalled that there exists a positive feedback mechanism since many of the secreted factors can stimulate NF-κB signaling via their specific receptors both in immune and nonimmune cells. Moreover, NF-κB signaling has many interactions with metabolic systems and other signaling pathways which act in cooperation to establish an inflammatory regulatory network (Oeckinghaus et al. 2011; Platanitis and Decker 2018; Capece et al. 2022). It is known that chemokines and CSFs are the most common SASP components secreted by diverse types of senescent cells in different experimental models (Freund et al. 2010). The activation of cGAS-STING signaling induces a strong expression and secretion of several chemokines in many experimental conditions (Hao et al. 2022; Withers et al. 2023; Talbot et al. 2024). Both the NF-κB and IRF3 signaling pathways are able to stimulate the expression of chemokines which means that cGAS-STING signaling can promote chemotaxis of myeloid and lymphoid cells and their recruitment into inflamed tissues and thus enhance inflammatory state. Moreover, the STING factor itself and NF-κB signaling can stimulate proinflammatory responses by activating NLRP inflammasomes through different mechanisms (Bauernfeind et al. 2009; Wang et al 2020) (Fig. 1). The production of IL-1β and IL-18 cytokines promotes the proinflammatory state within the tissues.

Type-1 IFNs are important SASP factors which evoke diverse immune responses through the regulation of many ISGs via the IFNAR/JAK1/STAT1/2 signaling pathway (Schneider et al. 2014; Ji et al. 2023). It is known that the ISGs represent about 10% of the human genome and type-1 IFNs control the transcription of as many as 450 genes (Schoggins 2019). The major function of STING/IRF3/IFN signaling is to control the antiviral responses although this axis is a pleiotropic pathway which can regulate not only the innate and adaptive immunity but also generate alterations in cell fates, e.g., induce apoptosis and cellular senescence as well as triggering immunogenic cell death (ICD), such as ferroptosis, necroptosis, and pyroptosis (Murthy et al. 2020; Hu et al. 2022; Kroemer et al. 2022) (Fig. 2). Type-1 IFNs have a crucial role in different inflammatory conditions, e.g., macrophages use IFN signaling to augment inflammatory responses in autoimmune diseases although an overactivity of type-1 IFN signaling leads to interferonopathies (Siebeler et al. 2023; Mendonca and Fremond 2024). The therapeutic role of type-1 IFNs in viral infections, cancers, and inflammatory diseases is attributable to their antiproliferative activities mediated via the STAT1/2 pathway (Bromberg et al. 1996; Bekisz et al. 2010). Moreover, Popli et al. (2022) demonstrated that the IRF3 protein inhibited NF-κB signaling in the cytoplasm of human cells and thus suppressed the viral-activated NF-κB inflammatory response. Recently, Zannikou et al. (2024) reviewed the specific functions of type-1 IFNs in different immune cells and emphasized their context-dependent responses in common immune diseases.

## cGAS-STING signaling remodels hematopoiesis and thymic involution

There is abundant evidence that the aging process affects the functions of hematopoietic stem and progenitor cells (HSPC) in the bone marrow (BM) (Elias et al. 2017; Gnani et al. 2019; Ho et al. 2019). It is known that clonal selection with aging supports an expansion of myeloid lineages in both mice and humans (Elias et al. 2017; Ho et al. 2019). Interestingly, the aging process is associated with an accumulation of senescent cells and the occurrence of inflammatory microenvironment within the BM (Hellmich et al. 2023). Many investigators have revealed that DNA damage and chronic inflammation are the major sources of cellular senescence in the BM (Moehrle and Geiger 2016; Fujino et al. 2022). One possible source could be many pro-inflammatory SASP factors which are released from senescent cells present in aged peripheral tissues (Fujino et al. 2022; Bogeska et al. 2022). On the other hand, HSCs are susceptible to age-related genotoxic stress and thus genetic lesions could become propagated via separate lineages into different hematological pools. The components of cGAS-STING signaling are widely expressed in the hematopoietic system, especially in HSPCs (Liao et al. 2020). cGAS-STING signaling has an important role in the regulation of hematopoiesis both in steady-state and especially in pathological conditions (Liao et al. 2020). cGAS-STING signaling controls several functions of HSPCs via the regulation of IRF3 and NF-κB signaling. For example, systemic administration of a STING agonist in mice promoted myeloid cell maturation and increased myelopoiesis (Xu et al. 2023). Especially, type-1 IFNs have a crucial role in the regulation of HSCs and hematopoiesis. Interestingly, type-1 IFNs produced by bacterial microbiota in the gut can regulate hematopoiesis in mice (Yan et al. 2022). This could explain why antibiotic therapy can induce adverse hematological responses, such as cytopenia. There are several studies indicating that the aging process affects hematopoiesis, e.g., it robustly increases myelopoiesis that maintains an inflammatory state (Beerman et al. 2010; Elias et al. 2017). Given that aging is associated with DNA damage and senescence in the cells of the BM, it seems that cGAS-STING signaling may promote the aging process via remodelling the functions of HSPCs.

An involution of the thymus is a crucial hallmark of the aging process (Thomas et al. 2020; Liang et al. 2022). In particular, an age-related degeneration appears in thymic stromal epithelial cells which have a major role in the maturation of T cells. It seems that thymic involution contributes to immunosenescence and thus could also aggravate the process of inflammaging (Thomas et al. 2020). It is known that not only immunosenescence but also cellular senescence and inflammation are involved in human thymic involution (Sato et al. 2017; Barbouti et al. 2019). The STING protein is highly expressed in the endothelial cell compartment, T cells, and macrophages in human thymus (Human Protein Atlas). The STING protein is mainly expressed in human medullary thymic epithelial cells and its activation stimulates the expression of type-1 IFNs (Deng et al. 2025). Excessive IFN signaling causes defects in the maturation of T cells and thus contributes to autoimmunity. Furthermore, an experimentally-induced increase in cytosolic dsDNA in mouse thymus stimulates the cGAS-STING-mediated apoptosis of thymocytes thus impairing T cell development (Ratiu et al. 2022). IFN-α is constitutively expressed in human thymus where it regulates the differentiation of T cells (Colantonio et al. 2011). The IFN-α factor is also a crucial mediator of pathogen-induced thymic involution in mice (Papadopoulou et al. 2011). Given that infections are major inducers of cGAS-STING signaling, it seems that STING/type-1 IFN signaling has an important role in thymic involution and immunosenescence with aging.

## cGAS-STING signaling promotes immunosuppressive responses

The cGAS-STING pathway is a pleiotropic immune regulator since it stimulates not only proinflammatory responses but it can also evoke anti-inflammatory and immunosuppressive responses, e.g., in the resolution phase after an acute inflammation as well as in many chronic inflammatory states (Lemos et al. 2014; Liang et al. 2017; Damasceno et al. 2022; Chen et al. 2024) (Fig. 2). For instance, Chen et al. (2024) demonstrated that during wound healing cGAS-STING signaling induced a systemic mobilization of myeloid cells into wounded skin. STING signaling promoted chemokine production by macrophages which regulates monocyte chemotaxis from the BM via the CCL2/CCR2 chemokine axis. They also reported that the knockout of STING significantly delayed the resolution of inflammation in wounded skin. There is clear evidence that the activation of STING/type-1 IFN signaling stimulates the recruitment of immunosuppressive M-MDSCs via the CCL2/CCR2 pathway into mouse tumors (Liang et al. 2017) and multiple sclerotic lesions (Melero-Jerez et al. 2019). Moreover, a chronic exposure of mice to type-1 IFNs can significantly elevate the level of MDSCs in the circulation and in the spleen (Taleb et al. 2017). There are also observations indicating that IFN-β exposure can suppress inflammation by inhibiting the adhesion and infiltration of leukocytes through the microvascular endothelium in human brain (Lou et al. 1999; Creange et al. 2001). It seems that an activation of cGAS-STING signaling and the subsequent secretion of type-1 IFNs can stimulate immune suppressive responses in inflamed tissues, probably in a context-dependent manner.

The secretion of SASP factors not only contains proinflammatory factors but there are many proteins which enhance anti-inflammatory and immunosuppressive responses. For instance, IL-10 and GDF15 are the core components of the SASP secretion in different experimental models (Basisty et al. 2020; Fan et al. 2024; Salminen 2025a). The IL-10 cytokine is an interesting SASP factor since it can enhance cellular senescence in non-immune cells as well as promoting immunosuppression in many immune cells, such as MDSCs, Tregs, and macrophages (Levings et al. 2001; Ouyang et al. 2011). It is known that STING/type-1 IFN signaling stimulates the expression and secretion of IL-10 in different cell types (Ziegler-Heitbrock et al. 2003; Ahn et al. 2017; Field et al. 2020). IL-10 is a potent suppressor of proinflammatory T helper 17 cells (Th17) (Stewart et al. 2013; Guo 2016). Th17 cells have an important role in the pathogenesis of many autoimmune diseases, e.g., psoriasis, rheumatoid arthritis, and multiple sclerosis (Tesmer et al 2008). Interestingly, Damasceno et al. (2022) demonstrated that STING signaling is an intrinsic checkpoint inhibitor in mouse Th17 cells limiting their pathogenic responses. They reported that an activation of STING signaling increased the expression of IL-10, whereas it reduced the expression of IL-17, a proinflammatory cytokine secreted by Th17 cells. In fact, the Th17 and Treg cells, both are the subsets of CD4^+^ T cells, establish an immune balance in autoimmune diseases, i.e., Th17 cells promote inflammation and autoimmunity, whereas Treg cells exert opposite effects and maintain immune homeostasis (Lee 2018). It seems that an activation of the STING pathway can regulate the balance between Treg and Th17 cells via type-1 IFN/IL-10 signaling switching the equilibrium towards immunosuppressive responses.

The immunosuppressive network involves both immunosuppressive cells, such as MDSCs, Tregs, and M2 macrophages, and many inhibitory immune checkpoint receptor/ligand systems. There are diverse inhibitory checkpoint receptors, especially in surveying cytotoxic CD8^+^ T cells and natural killer (NK) cells which drive immune elimination of invading pathogens as well as aberrant host cells, such as senescent and tumor cells (Zhang and Zheng 2020; Baldanzi 2022; Wang et al. 2022). The programmed cell death protein-1 (PD-1) and its death-ligand 1 (PD-L1) establish the best characterized inhibitory checkpoint axis which for example, suppresses the activation of T and NK cells and thus control many of the functions of the immune system. Interestingly, it is known that cGAS-STING signaling stimulates the expression of the PD-L1 protein in several cell types and experimental models (Du et al. 2022; Kim et al. 2024; Shen et al. 2024). Many investigators have reported that cGAS-STING signaling is able to stimulate the expression of PD-L1 either through the extrinsic pathway of IFN/IFNAR1/STAT1 or directly via the STING-stimulated IRF3 transcription factor (Schreiner et al. 2004; Xiao et al. 2018; Du et al. 2022). Interestingly, recent studies have revealed that the expression of the PD-L1 protein is significantly increased in senescent cells induced by diverse insults (Onorati et al. 2022; Wang et al. 2022; Majewska et al. 2024; Salminen 2024a). Wang et al. (2022) revealed that an increase in the expression of PD-L1 in mouse senescent cells prevented their immunosurveillance both in cell culture and in aged tissues. Currently, it is known that cellular senescence is associated not only with an increase in PD-L1 expression but also with many other ligand proteins of inhibitory checkpoint receptors, such as those of the LILRB4, NKG2A, TIM-3, and SIRPα pathways (Salminen 2024b). Paradoxically, it seems that senescent cells can prevent their own immune surveillance and concurrently, they suppress the activation of many immune cells in aged tissues and thus promote immunosenescence and the aging process (Salminen 2025b).

cGAS-STING signaling seems to have double-edged effects on the regulation of the immunosuppressive microenvironments in tumors (Li and Bakhoum 2022; Zhang et al. 2024). As described above, an activation of cGAS-STING signaling in tumor cells stimulates the expression of many ligands of the inhibitory immune checkpoint receptors, such as PD-L1, and thus provides a route for immune evasion by tumor cells. Moreover, cGAS-STING signaling stimulates the expression of many chemokines which recruit immunosuppressive cells, such as MDSCs and Tregs, into the tumor microenvironment which subsequently promote metastasis (Liang et al. 2017; Nambiar et al. 2023). On the other hand, Zhang et al. (2019) demonstrated that STING-TBK1 signaling suppressed the differentiation of MDSCs by enhancing SOCS1 expression which inhibited STAT3 signaling, a master pathway of immunosuppressive responses. Accordingly, Cheng et al. (2020) revealed that cGAMP exposure suppressed the activity of MDSCs and inhibited tumor growth and metastasis in mice, whereas a STING deficiency increased metastasis of murine colon cancer cells. Currently, it seems that there are opposite effects whether cGAS-STING signaling is activated in immune cells or tumor cells.

In contrast to MDSCs, it seems that cGAS-STING signaling not only induce the infiltration of Treg cells into target tissues but it can also stimulate the differentiation and promote immunosuppressive properties of Tregs (Liang et al. 2015; Ni et al. 2022; Eskandari et al. 2023; Lin et al. 2024). There are observations indicating that cGAS-STING signaling induces the differentiation of Tregs not only via the IRF3/type-1 IFN pathway but also through IFN-independent signaling, e.g., via the SMAD3 and STAT5 pathways (Ni et al. 2022) or CREB signaling (Lin et al. 2024) in a context-dependent manner. However, type-1 IFNs can induce the polarization of mouse T cells into FOXP3-positive Tregs and enhance their immunosuppressive activity in different experimental conditions (Lee et al. 2012; Vitale et al. 2021; Eskandari et al. 2023). In conclusion, there is abundant evidence that cGAS-STING signaling has crucial effects on the functions of the immune network although it appears that the responses are specific and dependent on the pathological state of the tissues.

## cGAS-STING signaling promotes the aging process

An increase in the cytosolic level of dsDNA is an important hallmark of the aging process (Lopez-Otin et al. 2023). The theory that DNA damage underpinned the aging process was presented over fifty years ago (Alexander 1967). Later, it was hypothesized that transposons could promote the aging process (Murray 1990). The mitochondrial theory of aging has encompassed many hypotheses during the past years, such as free radical production, unfolded protein responses (UPRmt), and a decay in mitochondrial energy metabolism. More recently, it was observed that the aging process was associated with a leakage of mtDNA which is a damage-associated molecular pattern (DAMP) recognized by dsDNA sensors (Newman and Shadel 2023; Jimenez-Loygorri et al. 2024). In fact, these age-related molecular changes in tissues are also present in senescent cells which indicates that they originate in senescent cells and accumulate with aging. It seems that an increase in the amount of cytosolic dsDNA is a driving force exerting pathological processes which are propelled by dsDNA sensors, especially by cGAS-STING signaling via many innate and adaptive immune responses. It is not only non-immune cells which display an accumulation of senescent cells with aging but many immune cells also undergo alterations reminiscent of those occurring in cellular senescence (Xu and Larbi 2017; Salminen 2021; Mogilenko et al. 2022). Interestingly, mitochondrial dysfunctions with a leakage of dsDNA have been observed with aging in many immune cells, e.g., T cells, macrophages, and microglia (Escrig-Larena et al. 2023; Maurmann et al. 2023; Liu et al. 2024). An age-related defective mitophagy seems to be the reason for an increased leakage of mtDNA in senescent cells (Jimenez-Loygorri et al. 2024). It seems that the accumulation of cytosolic dsDNA with aging is a cellular checkpoint to alert the immune system that there is a risk that tissue homeostasis will be disturbed.

There are several investigations indicating that the cGAS-STING sensor pathway not only promotes cellular senescence but it can also remodel the immune network and thus aggravate many age-related diseases (Fig. 2). Gulen et al. (2023) demonstrated that an activation of the cGAS-STING pathway was a crucial driver of chronic low-grade inflammation with aging in many peripheral tissues and promoted functional decline in mice. They reported that inhibition of STING signaling with the selective H-151 inhibitor suppressed inflammatory responses in senescent cells and in many tissues of aged mice. They also reported that old STING knockout mice displayed a reduced level of microglial cells in the hippocampal area, whereas the density of neurons was survived better than in their aged counterparts. A histochemical assessment revealed that microglial cells in the hippocampus of normal aged mice were enriched with the expression of activated STING (pSTING) protein in comparison to their young counterparts. The isolated microglial cells from aged mice robustly secreted more immunoreactive proteins, such as those of interferon-stimulated genes (ISG), than microglial cells from control mice. Interestingly, Gulen et al. (2023) reported that an increase in intrinsic cGAS-STING signaling of microglial cells was accompanied by IFN-related transcriptomic alterations in astrocytes and oligodendrocytes but not in neurons. This seems to indicate that cGAMP release from aged microglia can stimulate cGAS-STING signaling in neighbouring cells and with aging this could promote neurodegeneration in the brain.

However, there are studies indicating that the canonical cGAS-STING signaling pathway mediated by the production of cGAMP is not the only pathway which promotes cellular senescence and the aging process. For instance, Cancado de Faria et al. (2025) demonstrated that although the cGAS and STING proteins were required for the sterile inflammatory state of senescent and progeria cells, which was driven by non-canonical pathway in a cGAMP-independent manner. Moreover, they reported that the activation of cGAS-STING signaling by dsDNA exposure declined in senescent/progeria cells. Cancado de Faria et al. (2025) also revealed that the inhibition of STING signaling in the *Lmna* progeria mice with H151 treatment significantly delayed cellular aging process, slowed tissue degeneration, and extended mouse lifespan. It is known that there are certain STING-activating signaling pathways which do not require cGAS-generated cGAMP production, i.e., the high mobility group box 1 (HMGB1) and poly (ADP-ribose) polymerase (PARP)/ataxia telangiectasia mutated (ATM) pathways (Dunphy et al. 2018; Lee et al. 2021). Both of these pathways are known to be triggered by genomic instability and subsequently they promote cellular senescence and the aging process (Zhao et al. 2020; Salminen 2026). Interestingly, there are recent reports indicating that the nuclear cGAS protein can maintain DNA stability and be involved in the DNA repair processes (Chen et al. 2020; Zhen et al. 2023; Zierhut 2024). Interestingly, Chen et al. (2025) demonstrated that the cGAS protein of the naked mole rats, living over thirty years, displayed alterations in four amino acids which enabled the cGAS protein to retain better chromatin structure in DNA damage by reducing the TRIM41-mediated ubiquitination. This property of the cGAS protein also enhanced the repair process of DNA damage. Given that DNA instability is a hallmark of the aging process, it seems that the cGAS protein has a dual role in the aging process, (i) it can first stabilize chromatin structure and promote DNA repair at a cellular level but later (ii) it stimulates immune responses via cGAS-STING signaling and thus promotes cellular senescence and the aging process.

There is convincing evidence that a decrease in mitophagy with aging increases the release of mtDNA from mitochondria and stimulates cGAS-STING signaling (Zhong et al. 2022; Ha et al. 2023; Jimenez-Loygorri et al. 2024; Zhou et al. 2024). Jimenez-Loygorri et al. (2024) demonstrated that cytosolic mtDNA stimulated a cGAS-STING-driven type-1 IFN response and aggravated inflammation in the retina of aged mice as well as in human dermal fibroblasts isolated from elderly donors. They reported that the pharmacological activation of mitophagy by a long-term treatment with urolithin A decreased the level of cytosolic mtDNA and reduced the expression of IFN-α and TNF-α in the retina of old mice. The urolithin A treatment also decreased the extent of astrogliosis in the retina of old mice. The PTEN-induced kinase 1 (PINK1) has a key role in the mitophagy and thus it controls the release of mtDNA and the activation of cGAS-STING signaling (Ha et al. 2023; Zhou et al. 2024). Zhou et al. (2024) revealed that the PINK1 knockout mice displayed in cardiac muscle (i) a defect in mitophagy, (ii) an increase in cytosolic mtDNA, and (iii) a robust activation of cGAS-STING signaling. A deficiency of the PINK1 protein induced inflammation in mouse myocardium and promoted cardiac hypertrophy, whereas the overexpression of the cardiac-specific PINK1 protein inhibited cGAS-STING signaling and prevented cardiac hypertrophy. Accordingly, the activation of cGAS-STING signaling in the PINK1 knockout mice promoted the aging process in the kidney by increasing the extent of cellular senescence as well as the severity of renal fibrosis and tubular injury (Ha et al. 2023). Moreover, there are observations indicating that cGAS-STING signaling is activated in the macrophages of aged mice due to an increased leakage of mtDNA into the cytoplasm (Zhong et al. 2022). These studies indicate that the functional properties of mitochondria decline with aging and the release of mtDNA into the cytoplasm stimulates cGAS-STING-driven cellular senescence which promotes the aging process in tissues. These results support the mitochondrial hypothesis of the aging process.

Disturbances in the integrity of nuclear membrane enhance the release of genomic fragments into the cytoplasm and subsequently trigger cGAS-STING signaling (Kreienkamp et al. 2018; Sladitschek-Martens et al. 2022; Yang et al. 2024). A loss of the integrity of the nuclear envelope not only triggers cellular senescence but it has also been reported to accelerate the aging process (Martins et al. 2020; Rocha et al. 2022). The Hutchinson-Gilford progeroid syndrome, a premature aging disease, is caused by aberrant splicing of the *LMNA* gene which generates mutant forms of the lamin protein, generally called progerins, which disturb the integrity of the nuclear envelope (Gonzalo et al. 2017). Sladitschek-Martens et al. (2022) demonstrated that the transcriptional coactivators of the Hippo pathway, i.e., Yes-associated protein (YAP) and transcriptional coactivator with PDZ-binding domain (TAZ) (YAP/TAZ signaling), played a crucial role in the control of the integrity of the nuclear envelope and subsequently regulated the aging process via cGAS-STING signaling. Briefly, they reported that with aging, the function of YAP/TAZ signaling significantly declined in stromal cells of mouse dermis. By applying the knockout technique for YAP and TAZ expression, they revealed that a loss of YAP/TAZ signaling robustly provoked the aging phenotype in mouse skin, e.g., cellular senescence, aberrant collagen deposition, and reduced the density of hair follicles. In contrast, the overexpression of the YAP protein in normal mice delayed or suppressed the aging features in mouse skin. Interestingly, they observed that YAP/TAZ signaling in skin fibroblasts inhibited cGAS/STING signaling as well as preventing signs of cellular senescence, such as the SASP phenotype. Sladitschek-Martens et al. (2022) addressed the question of how YAP/TAZ signaling could suppress cGAS-STING activity and delay the aging process. They revealed that in diverse human and mouse primary cells, YAP/TAZ signaling stimulated the expression of the lamin B1 and ACTR2 proteins and prevented a loss of F-actin caps in the nuclear envelope. This corroborated their observation that YAP/TAZ-depleted cells displayed a fragile nuclear envelope. These results opened a scenario where YAP/TAZ signaling inhibited the activation of cGAS-STING signaling and thus suppressed the senescence/aging process by inhibiting the fragmentation and release of genomic structures into the cytoplasm.

Kreienkamp et al. (2018) demonstrated that in human fibroblasts the expression of progerin, a lamin protein fragment, activated cGAS-STING signaling which stimulated a STAT1-mediated IFN response involving many ISG proteins. They also reported that STAT1/IFN signaling induced by progerin stimulated the expression of the cGAS, STING, and IRF3 proteins confirming a well-known positive feedback response in IFN signaling. There is clear evidence that increases in systemic and local IFN responses are not only associated with progerin pathology but they are also present in cellular senescence and the aging process in normal mice (Kreienkamp et al. 2018; Benayoun et al. 2019; Cao 2022; Rasa et al. 2022). Benayoun et al. (2019) demonstrated that different immune response pathways were significantly upregulated with aging in many murine tissues. Interestingly, the most robust age-related enrichment appeared to take place in the genes regulated by the IFN-α pathway. Rasa et al. (2022) reported that dietary restriction, a well-known antiaging modulator, notably inhibited the age-related IFN signatures and ameliorated the inflammaging process. In conclusion, there is mounting evidence that an accumulation of dsDNA into senescent cells can stimulate cGAS-STING signaling which subsequently promotes cellular senescence and the aging process by remodeling the properties of the immune network via the interferon and NF-κB pathways.

## Conclusions

There is a general consensus that cellular senescence has a crucial role in the progress of the aging process within tissues. It is also known that the proinflammatory SASP state in tissues as well as the inflammaging process promote the age-related degeneration of tissues. However, there are many mechanisms which can induce cellular senescence and promote the inflammaging process. Recently, many investigators have emphasised the role of dsDNA release into cytoplasm and the activation of signaling pathways driven by DNA sensors, such as the cGAS-STING pathway. Although the primary cause of dsDNA release from mitochondria and nuclei with aging still needs to be clarified, senescent cells themselves are able to remodel the immune network and accelerate the aging process. For instance, canonical cGAS-STING signaling is a potent driver of immune alterations which can lead to cellular senescence and tissue degeneration with aging. However, it seems that there are several non-canonical pathways associated with either the functions of nuclear cGAS protein or signaling pathways driven by the STING protein without cGAS activation. Especially the role of the cGAS protein in the maintenance of chromatin stability is an important research area. It is known that loss of chromatin integrity with aging leads not only to the release of cGAS but also HMGB1 which is an alarming factor activating the immune network and promoting cellular senescence (Salminen 2026). It seems that both cGAS and HMGB1 are nuclear alarmins which can promote the SASP state and trigger cellular senescence. Recent studies have revealed that the STING protein can also be translocated into nuclei where it can regulate transcription and control the functions of innate immune system (Dixon et al. 2021; Zhang et al. 2023; Wang et al. 2025). Dixon et al. (2021) reported that the STING protein was located in the inner nuclear membrane pool where it assembled an interactome with many nuclear membrane proteins. Currently, it is not known whether the STING protein is involved in the age-related disturbances in the integrity of nuclear membrane. It seems that non-canonical cGAS-STING processes have a crucial role in cellular and organismal aging, as revealed by recent investigations (Cancado de Faria et al. 2025; Chen et al. 2025).

## Data Availability

No datasets were generated or analysed during the current study.
